# Core 2 mucin-type *O*-glycan inhibits EPEC or EHEC O157:H7 invasion into HT-29 epithelial cells

**DOI:** 10.1186/s13099-015-0078-9

**Published:** 2015-12-15

**Authors:** Jun Ye, Qiong Pan, Yangyang Shang, Xiaolong Wei, Zhihong Peng, Wensheng Chen, Lei Chen, Rongquan Wang

**Affiliations:** Department of Gastroenterology, Southwest Hospital, Third Military Medical University, Chongqing, 400038 People’s Republic of China

**Keywords:** Mucin, *O*-glycan, Invasion, Enteropathogenic *E. coli*, Enterohemorrhagic *E. coli* O157:H7

## Abstract

**Background:**

How host cell glycosylation affects EPEC or EHEC O157:H7 invasion is unclear. This study investigated whether and how *O*-glycans were involved in EPEC or EHEC O157:H7 invasion into HT-29 cells.

**Results:**

Lectin histochemical staining confirmed stronger staining with PNA, which labeled Galβ1, 3 GalNAc (core 1 structure) in HT-29-Gal-OBN and C2GnT2-sh2/HT-29 cells, compared with control cells. EPEC or EHEC O157:H7 invasion into HT-29 and its derived cells was based on the intracellular presence of GFP-labeled bacteria. The differentiation of HT-29 cells led to a reduction in EPEC internalization compared with HT-29 cells (p < 0.01). EPEC or EHEC O157:H7 invasion into HT-29-OBN and HT-29-Gal-OBN cells increased compared with HT-29 and HT-29-Gal cells (p < 0.05 and p < 0.01). Core 2 *O*-glycan-deficient HT-29 cells underwent a significant increase in EPEC (p < 0.01) or EHEC O157:H7 (p < 0.05) invasion compared with control cells.

**Methods:**

Bacterial invasion into cultured cells was determined by a gentamicin protection assay and a GFP-labeled bacteria invasion assay. *O*-glycans biosynthesis was inhibited by benzyl-α-GalNAc, and core 2 *O*-glycan-deficient HT-29 cells were induced by C2GnT2 interference.

**Conclusion:**

These data indicated that EPEC or EHEC O157:H7 invasion into HT-29 cells was related to their *O*-glycosylation status. This study provided the first evidence of carbohydrate-dependent EPEC or EHEC O157:H7 invasion into host cells.

## Background

**Fig. 1:**
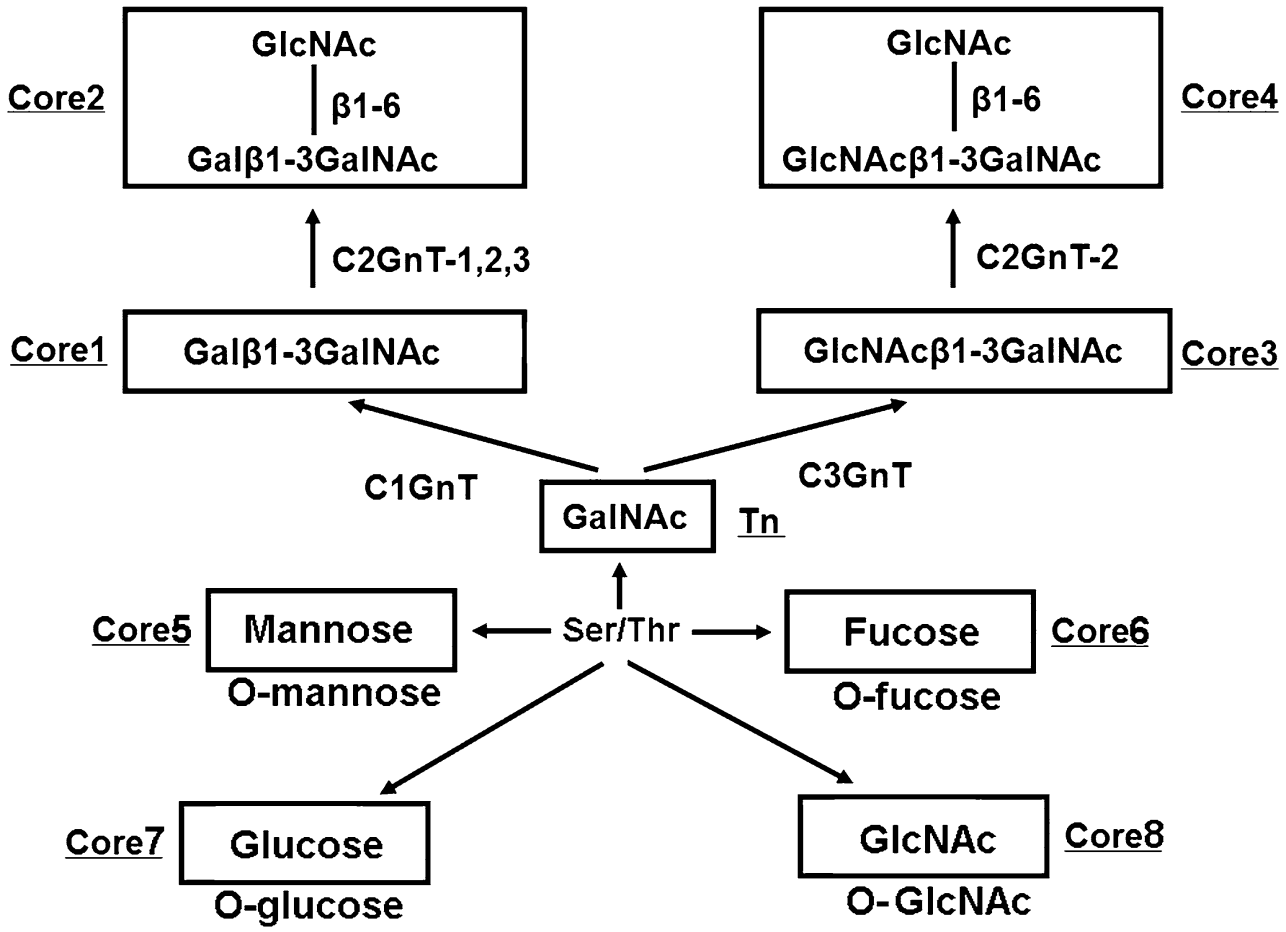
Illustration of mucin-type O-glycan synthesis. Starting from N-acetylgalactosamine (GalNAc) on serine or threonine residues in a polypeptide, core 1 synthase (C1GnT) transfers galactose to make the Core 1 structure Galβ1 → 3GalNAcα1 → Ser/Thr, and core 3 synthase (C3GnT) transfers N-acetylglucosamine to form a core 3 structure GlcNAcβ1 → 3GalNAcα1 → Ser/Thr (Core 3). Core 1 is converted to Core 2 by C2GnT-1, C2GnT-2, and C2GnT-3. Core 3 is converted to Core 4 by C2GnT-2. Mannose, fucose, glucose and GlcNAc are directly linked to Ser/Thr, and they form core 5-8 structures

The mucin layer functions as a barrier to gastrointestinal tract (GI) bacterial infection, effectively hampering bacteria from adhering to and invading into cells [1, 2]. Mucin-type *O*-glycans are classified into eight major groups (cores 1–8) based on their different carbohydrate residues [1]. Mucin-type core 2 *O*-glycan is biosynthesized by the enzyme core 2 β1, 6-N-acetylglucosaminyltransferase 2 (C2GnT2) (Fig. 1), which is mainly expressed in the colon [3, 4].

Enteropathogenic *E. coli* (EPEC) and Enterohemorrhagic *E. coli* O157:H7 (EHEC O157:H7) cause illnesses from mild diarrhea to severe diseases, such as hemorrhagic colitis [5]. *O*-glycans might be related to the selection of the commensal flora in the distal colon, and it could act as an attachment site for different bacteria [6]. We have reported that core 2 *O*-glycan deficiency in HT-29 cells and enhanced MUC3 expression in HT-29-Gal cells resulted in decreased EPEC or EHEC O157:H7 adherence [7, 8].

A sub-population of EPEC or EHEC O157:H7 is able to enter cultured intestinal epithelial cells [9–12]. Intimate attachment and invasion by EPEC and EHEC O157:H7 are both mediated by a 94-kDa intimin, which is encoded by the *eae* gene [13]. There is a strong dependence on microfilaments, depolymerization of microtubules and receptor-mediated endocytosis in the uptake of EPEC or EHEC O157:H7 by intestinal epithelial cells, suggesting that bacterial invasion requires both bacterial and eukaryotic protein syntheses [10].

To invade the epithelium, microbes commonly interact with glycan structures of the host glycocalyx [14, 15]. Both EPEC and EHEC O157:H7 can bind the intestinal mucin and the glycosylated mucin, which are recycled between the plasma membrane and the trans-Golgi network (TGN) or Golgi complex [16]. It is possible that recycling of the glycosylated mucin results in internalization of the binding EPEC or EHEC O157:H7. This fact inspired us to investigate whether mucin-type *O*-glycans are involved in EPEC or EHEC O157:H7 internalization in intestinal epithelial cells. We identified the *O*-glycosylation status of HT-29-OBN cells and mucin-type core 2 *O*-glycan deficient HT-29 cells, using a series of lectin recognition: *Maackia amurensis* (MAA), *Arachis hypogaea* (PNA), *Dolichos biflorus* (DBA), *Ulex europaeus* (UEA-I), *Griffonia simplicifolia* (GSAII), *Canavalia ensiformis* (ConA), and *Sambucus nigra* (SNA), and we further demonstrated that core 2 mucin-type *O*-glycan inhibited EPEC or EHEC O157:H7 invasion into HT-29 cells.

## Results

### Benzyl-α-GalNAc-treated HT-29 or HT-29-Gal cells altered their *O*-glycosylation status

**Fig. 2:**
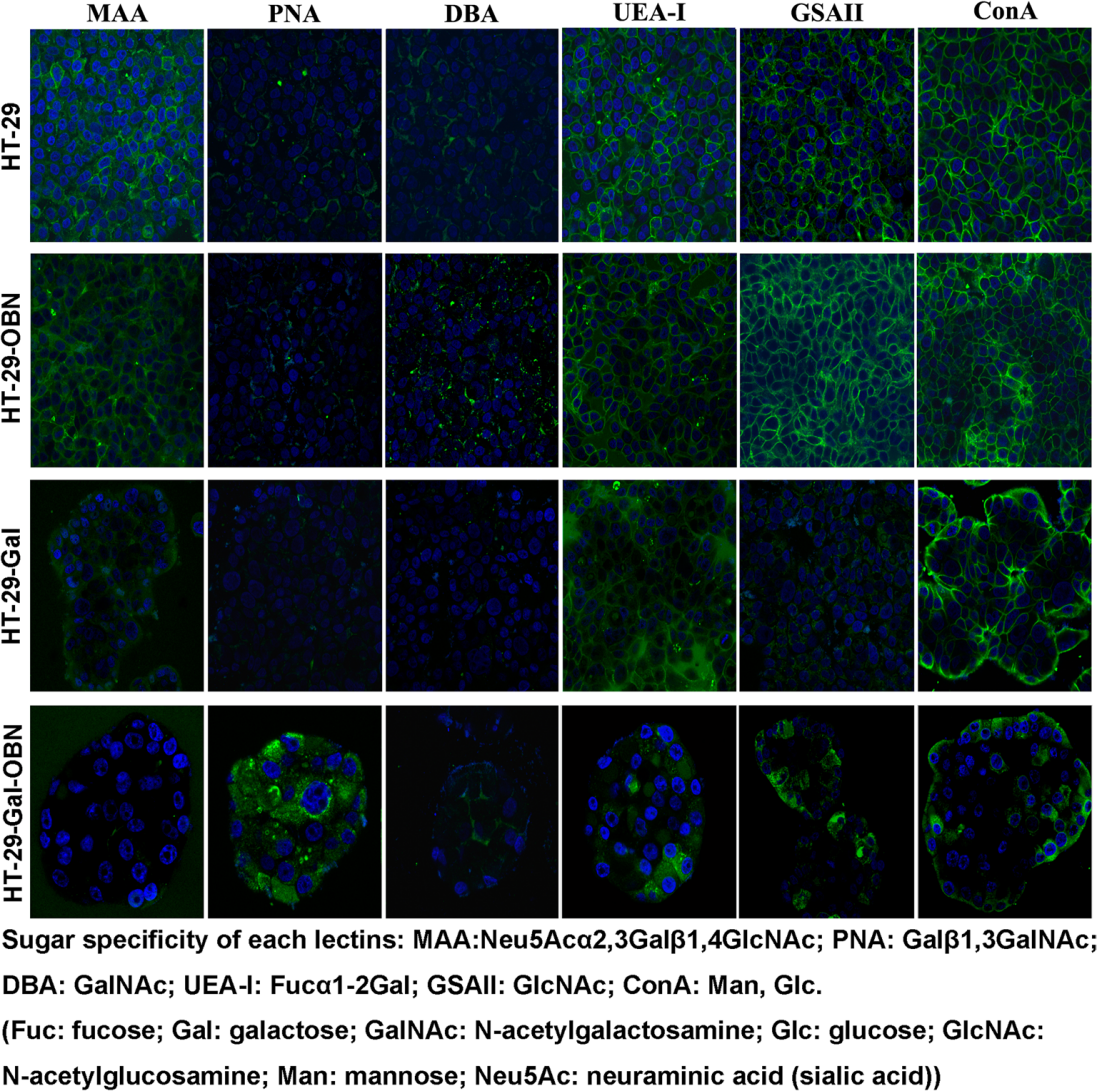
Lectin histochemical staining in HT-29, HT-29-OBN, HT-29-Gal, and HT-29-Gal-OBN cells. HT-29, HT-29-OBN, HT-29-Gal, or HT-29-Gal-OBN cells were stained with FITC-labeled MAA, PNA, DBA, UEA-I, GSAII or ConA lectins and were observed under fluorescence microscopy. ×40 objective

To confirm whether altered *O*-glycosylation occurred in HT-29-Gal, HT-29-OBN and HT-29-Gal-OBN cells [7], lectin histochemistry was performed using FITC-labeled MAA, PNA, DBA, UEA-I, GSAII or ConA. As shown in Fig. 2, similar lectin staining was found in HT-29 and HT-29-OBN cells. MAA, PNA, DBA and GSAII histochemical staining of HT-29-Gal cells was lower than that of HT-29 cells, indicating lower α-(2,3)-linked sialic acid terminated, core 1, GalNAc terminated and GlcNAc terminated structures in HT-29-Gal cells. PNA was increased, and MAA was reduced in HT-29-Gal-OBN cells compared to HT-29-Gal cells, indicating more core 1 structures and lower α-(2,3)-linked sialic acid-terminated structures (lower MAA staining) in HT-29-Gal-OBN cells. The spherical alignment of the HT-29-Gal and HT-29-Gal-OBN cells indicated that they possessed the differentiated phenotype.

### Direct evidence of GFP-labeled EPEC or EHEC O157:H7 invasion into intestinal epithelial cells

**Fig. 3:**
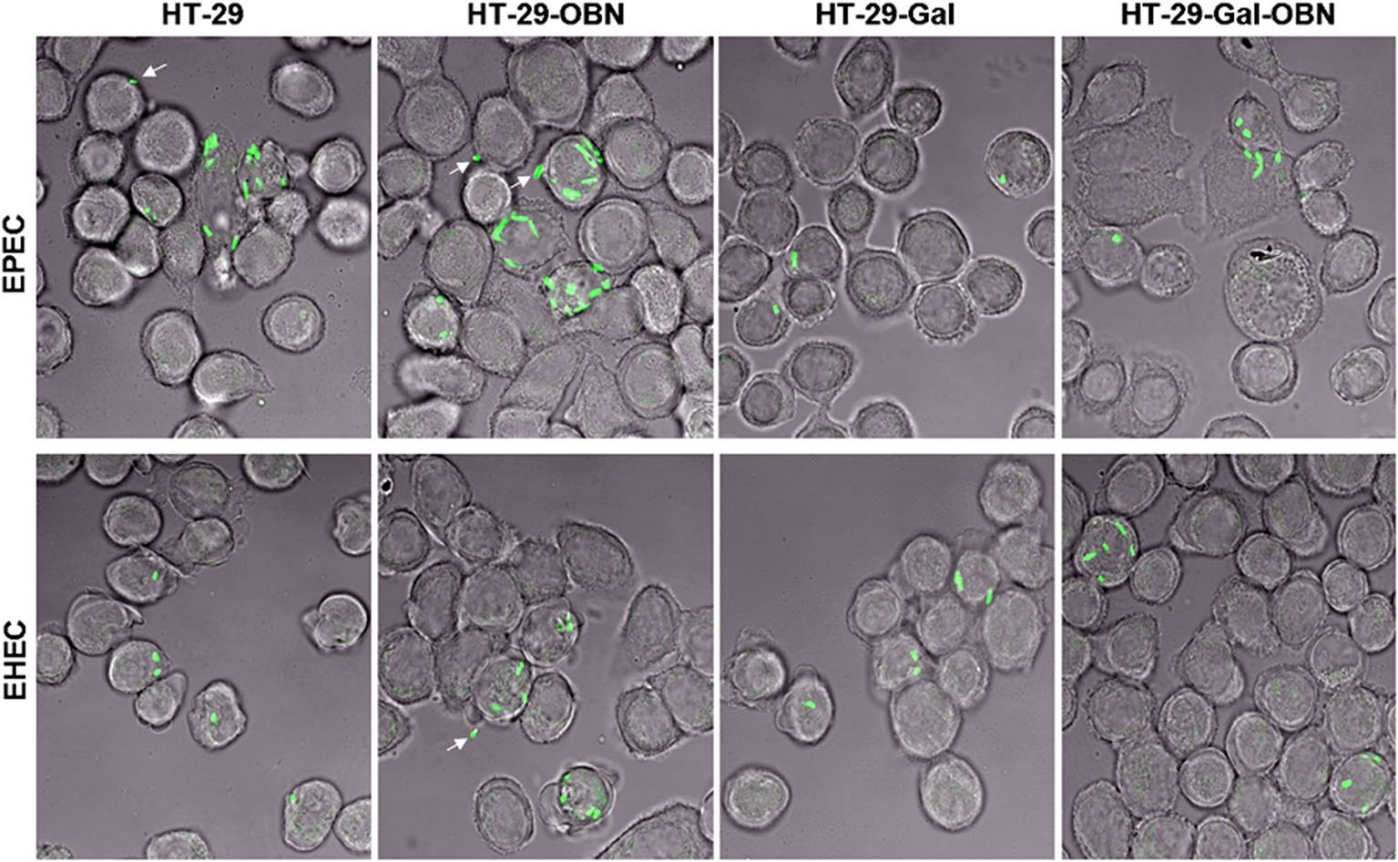
Identification of bacterial invasion with GFP-labeled EPEC or EHEC O157:H7. The internalized GFP-labeled EPEC or GFP-labeled EHEC O157:H7 within HT-29, HT-29-OBN, HT-29-Gal, or HT-29-Gal-OBN cells was observed under a confocal microscope after co-culturing with GFP-labeled bacteria and tissue cells and further gentamicin treatment. The internalized bacteria were confirmed based on the presence of green fluorescence within the bacteria-infected cells. ×63 objective

To confirm whether EPEC or EHEC O157:H7 could invade into HT-29, HT-29-OBN, HT-29-Gal, or HT-29-Gal-OBN cells, bacterial invasion experiments using GFP-labeled EPEC or EHEC O157:H7 in HT-29-Gal, HT-29-Gal-OBN, HT-29 or HT-29-OBN cells were observed under a confocal microscope after co-culturing with GFP-labeled bacteria and tissue cells and further gentamicin treatment. As shown in Fig. 3, the majority of GFP-labeled EPEC or EHEC O157:H7 was exactly located in the cytoplasm of some of the bacteria-infected HT-29, HT-29-OBN, HT-29-Gal, and HT-29-Gal-OBN cells, although some of the adherent GFP-labeled EPEC or EHEC O157:H7 was located on the cell surface (indicated by arrowhead). However, it was difficult to quantify the amount of internalized bacteria according to the green fluorescence-labeled bacteria using the confocal microscope. The traditional bacteria invasion assay was further used to quantify the internalized bacteria.

### HT-29 cell differentiation was related to EPEC or EHEC O157:H7 invasion

A traditional gentamicin invasion assay was used to quantify the numbers of invaded bacteria within the HT-29, HT-29-OBN, HT-29-Gal, or HT-29-Gal-OBN cells. Because the traditional gentamicin invasion assay requires bacterial sensitivity to gentamicin, the EPEC E2348/69 strain (serotype: O157:H7) (EPEC) and EHEC Sakai stain (serotype: O157:H7) (EHEC O157:H7) used in this study were checked for the susceptibility/resistance pattern of gentamicin, and neither of the bacteria could survive in 100 μg/ml gentamicin containing cell culture medium or could grow in 100 μg/ml gentamicin containing MacConkey agar with overnight incubation at 37 °C.

**Fig. 4:**
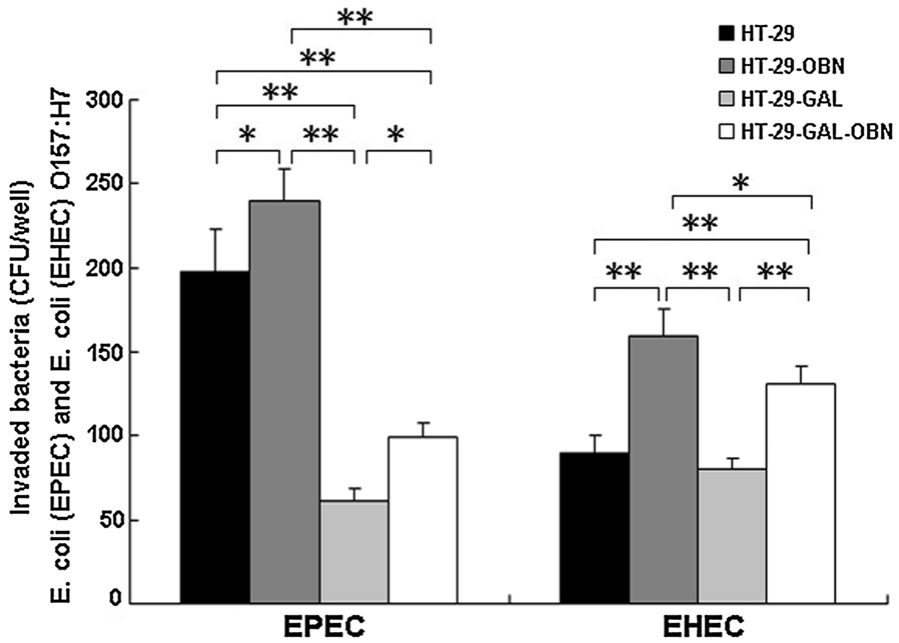
Comparison of EPEC or EHEC O157:H7 invasion into HT-29, HT-29-OBN, HT-29-Gal, and HT-29-Gal-OBN cells using bacteria invasion assay. HT-29, HT-29-OBN, HT-29-Gal, and HT-29-Gal-OBN cells were incubated with EPEC or EHEC O157:H7, the adherent bacteria were removed by gentamicin, and the internalized EPEC or EHEC O157:H7 within the different cells was quantified by determining the CFU following by the plating of serial dilutions of the bacteria on MacConkey agar with overnight incubation at 37 °C. *Asterisk* represents a significant difference (p < 0.05), *double asterisk* represents a highly significant difference (p < 0.01)

The number of EPEC bacteria that invaded into differentiated HT-29-Gal cells (61 ± 8) was significantly lower than that of EPEC bacteria invading into undifferentiated HT-29 cells (198 ± 25 CFU) (p < 0.01); the number of EHEC O157:H7 bacteria that invaded into differentiated HT-29-Gal cells (80 ± 7 CFU) was similar with that of EHEC O157:H7 bacteria invading into undifferentiated HT-29 cells (90 ± 10 CFU) (Fig. 4). The results indicated that the differentiation of HT-29 cells affected the amount of EPEC invasion into HT-29 cells, but it did not affect EHEC O157:H7 bacteria invasion.

### EPEC or EHEC O157:H7 invasion increased after the abrogation of *O*-glycan biosynthesis

The traditional bacteria invasion assay showed that the number of EPEC bacteria that invaded into the HT-29-Gal-OBN cells was 99 ± 9 CFU, which was significantly higher than the number that invaded the HT-29-Gal cells (61 ± 8) (p < 0.05), and the number of EHEC bacteria that invaded the HT-29-Gal-OBN cells was 131 ± 11 CFU, which was significantly higher than the number that invaded the HT-29-Gal cells (80 ± 7 CFU) (p < 0.01). The number of EPEC bacteria that invaded into the HT-29-OBN cells was 240 ± 19 CFU, which was significantly higher than the number that invaded the HT-29 cells (198 ± 25 CFU) (p < 0.05), and the number of EHEC bacteria that invaded into the HT-29-OBN cells was 160 ± 15 CFU, which was significantly higher than the number that invaded the HT-29-Gal cells (90 ± 10 CFU) (p < 0.01) (Fig. 4). These results indicated that the abrogation of *O*-glycan biosynthesis in the HT-29-Gal cells and HT-29 cells was beneficial for EPEC or EHEC O157:H7 invasion into cells.

To exclude the possibility in which the alteration of EPEC or EHEC O157:H7 invasion into the differentiated HT-29 cells and *O*-glycan biosynthesis inhibition of HT-29 cells was caused by their apoptosis, we performed apoptosis experiments (TUNEL assay) in HT-29, HT-29-Gal, HT-29-OBN and HT-29-Gal-OBN cells [7], and we found no significant difference in the apoptosis index among HT-29 (2.4 ± 0.3), HT-29-OBN (2.9 ± 0.7), HT-29-Gal (2.5 ± 0.8) and HT-29-Gal-OBN (1.8 ± 0.7) cells. Thus, the similarity of the apoptosis index among HT-29, HT-29-OBN, HT-29-Gal and HT-29-Gal-OBN cells indicated that the differentiation and the inhibition of *O*-glycosylation due to benzyl-α-GalNAc treatment in HT-29 cells did not cause their apoptosis compared with HT-29 cells, and the alteration of EPEC or EHEC O157:H7 invasion into the differentiated HT-29 cells and *O*-glycan biosynthesis inhibition of HT-29 cells were not caused by their apoptosis.

### C2GnT2-deficient HT-29 cells altered their *O*-glycosylation status

**Fig. 5:**
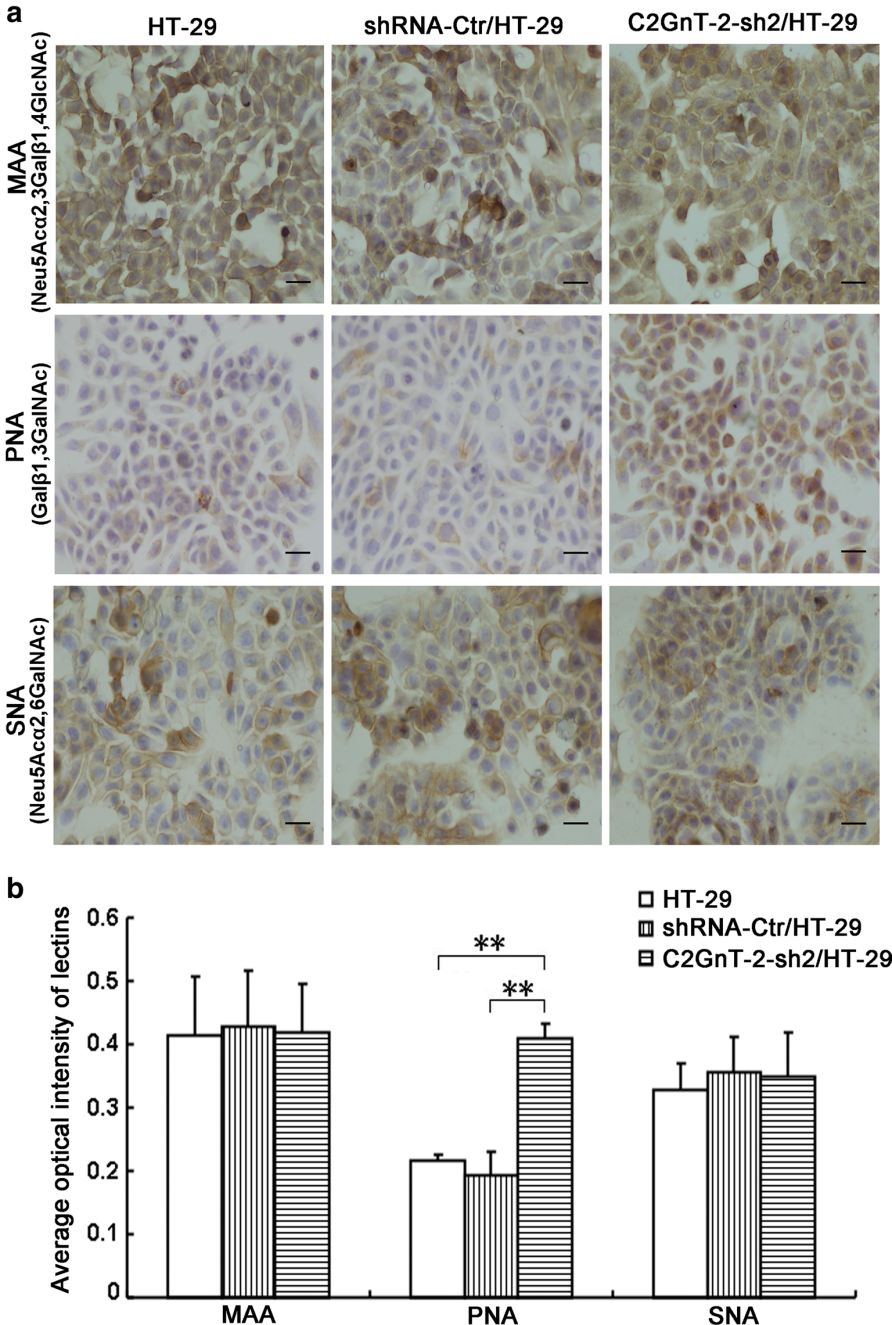
Lectin histochemical staining in HT-29, shRNA-Ctr/HT-29 and shRNA- C2GnT2/HT-29 cells. **a** HT-29, shRNA-Ctr/HT-29 and shRNA-C2GnT2/HT-29 cells were stained with the biotinylated MAA, PNA or SNA lectins and observed under light microscopy. **b** Analysis of average optical intensity of lectin staining (biotinylated MAA, PNA or SNA lectins) in HT-29, shRNA-Ctr/HT-29 or shRNA-C2GnT2/HT-29 cells. *Scale bars* 200 μm

To determine whether *O*-glycosylation status was altered in C2GnT2-deficient HT-29 cells, we chose biotin-labeled MAA, PNA or SNA to perform lectin histochemistry in HT-29, shRNA-Ctr/HT-29 and C2GnT2-sh2/HT-29 cells. MAA and SNA staining of the C2GnT2-sh2/HT-29 cells was similar to that of the HT-29 and shRNA-Ctr/HT-29 cells. PNA staining in the C2GnT2-sh2/HT-29 cells was significantly stronger than in the HT-29 and shRNA-Ctr/HT-29 cells (Fig. 5). Because PNA specifically binds to Galβ1,3GalNAc, which are *O*-glycan core 1 structures, it was consistent with the speculation that the *O*-glycan core 1 structure would be exposed due to the inhibition of *O*-glycan core 2 synthesis in C2GnT2-deficient HT-29 cells.

### C2GnT2-deficient HT-29 cells had more bacteria invasion

**Fig. 6:**
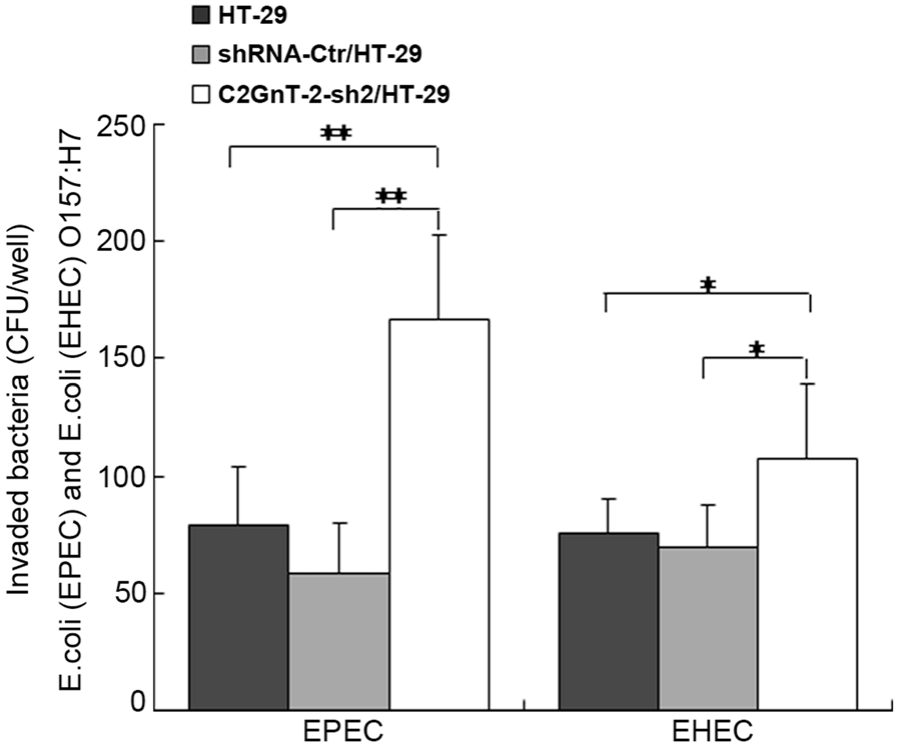
Comparison of EPEC or EHEC O157:H7 invasion into HT-29, shRNA-Ctr/HT-29 and shRNA-C2GnT2/HT-29 cells using bacteria invasion assay. HT-29, shRNA-Ctr/HT-29 and shRNA-C2GnT2/HT-29 cells were incubated with EPEC or EHEC O157:H7, the adherent bacteria were removed by gentamicin, and the internalized EPEC or EHEC O157:H7 within the different cells was quantified by determining their CFU. *Asterisk* represents a significant difference (p < 0.05), *double asterisk* represents a highly significant difference (p < 0.01)

HT-29, shRNA-Ctr/HT-29 and C2GnT2-sh2/HT-29 cells were used in a bacterial invasion assay. As shown in Fig. 6, 79 ± 25 and 58 ± 22 CFU of EPEC invaded the HT-29 and shRNA-Ctr/HT-29 cells, respectively, and 167 ± 36 CFU of EPEC invaded the C2GnT2-sh2/HT-29 cells. More EPEC invaded into the C2GnT2-sh2/HT-29 cells than into the HT-29 and shRNA-Ctr/HT-29 cells (p < 0.01). In addition, 76 ± 14 and 70 ± 18 CFU of EHEC O157:H7 invaded into the HT-29 and shRNA-Ctr/HT-29 cells, respectively, and 107 ± 32 CFU of EHEC O157:H7 invaded into the C2GnT2-sh2/HT-29 cells. More EHEC O157:H7 invaded into the C2GnT2-sh2/HT-29 cells than into the HT-29 and shRNA-Ctr/HT-29 cells (p < 0.05). These results indicated that EPEC or EHEC O157:H7 invasion into human HT-29 cells was related to the mucin-type core 2 *O*-glycan present in HT-29 cells.

Taken together, EPEC or EHEC O157:H7 invasion into HT-29 cells was related to their *O*-glycosylation status and differentiated stage; in particular, mucin-type core 2 *O*-glycan inhibited EPEC or EHEC O157:H7 invasion into HT-29 epithelial cells.

## Discussion

The internalization of EPEC or EHEC O157:H7 into cultured intestinal epithelial cells has been recognized with clear evidence. We confirmed here that mucin-type core 2 *O*-glycan might be one of the pivotal structures of *O*-glycosylated chains as protection mechanisms against EPEC or EHEC O157:H7 invasion.

EPEC intimin protein, type III secretion apparatus, EHEC O157:H7 CsgA and Lpp proteins and Curli fibers affect EPEC or EHEC O157:H7 internalization into eukaryotic cells [11, 17, 18]. Eukaryotic cells exert their own structural changes to adapt to EPEC or EHEC O157:H7 invasion. These cells belong to the “attaching and effacing (A/E) pathogen” category because of their ability to induce A/E lesions on intestinal epithelial cells; A/E lesions include localized effacement of the brush border microvilli, intimate bacterial attachment to the host epithelium, and the formation of cytoskeleton-rich pedestal structures beneath the adherent bacteria [19]. Intracellular EHEC O157:H7 were confirmed to be within membrane-bounded vacuoles by electron microscopy, EHEC O157:H7 invasion was dependent on eukaryotic microfilament assembly [20]. The effector EspT facilitated EPEC internalization into non-phagocytic cells in a process involving Rac1 and Wave2, which form intracellular actin pedestals [21].

Two useful models of bacterial invasion into eukaryotic cells are *Pseudomonas aeruginosa* and *Campylobacter jejuni.* Neuraminidase-1 (NEU-1)-dependent MUC1 ectodomain desialylation increased *Pseudomonas aeruginosa* invasion of airway epithelium [22]. Purified chicken intestinal mucin attenuated the adherence and invasion of *Campylobacter jejuni* in human HCT-8 cells in vitro, and this effect was attributed to mucin O-glycosylation [23]. *Campylobacter jejuni* invasion into HCT-8 cells was a glycan-mediated effect [24]. Crude human mucus tended to enhance *Campylobacter jejuni* binding and internalization [25].

EHEC O157:H7 invasion into human epithelial cells is known to be involved in some structures and processes of eukaryotic cells [10, 26]. The novel possibility of recycling-associated *O*-glycan processing from Gal1-4GlcNAc1-6(Gal1-3)GalNAc (core 2) to Gal1-3GalNAc (core 1) was found during the recycling of MUC1 [27]. Involvement of *O*-glycosylation in the intracellular trafficking of glycoproteins was found in polarized intestinal epithelial cells [28]. Using cell surface biotinylation and subcellular fractionation, increased accumulation of plasma membrane protein was found in endosomes after C1galt1 depletion. Confocal laser scanning microscopy and fluorimetry revealed increased translocation of negatively charged fluorescent nanospheres after C1galt1 knockdown, sustained by an active transport process [29]. Invasion of locus of enterocyte effacement (LEE)-positive and LEE-negative strains was higher for human enterocytic cell lines and for undifferentiated Caco-2 cells. Intracellular bacteria could be detected as early as 5 min after infection. Shiga toxin-producing *Escherichia coli* (STEC) invasion depended on actin microfilaments and protein kinases. Disruption of the tight junction occurred by EGTA-enhanced invasion of Caco-2 monolayers, and bacterial invasion mostly proceeded through the basolateral pole of enterocytes [30]. Pretreatment of HCT-8 cells with either the cholesterol-depleting agent methyl-β-cyclodextrin (MβCD) or the tyrosine kinase inhibitor genistein significantly decreased invasion by 98NK2, indicating a potential role for lipid rafts in the invasion mechanism [31]. The effect of hmLF glycosylation was examined for the protein’s ability to affect bacterial binding to epithelial cells. hmLF significantly inhibited pathogen adhesion, and purified hmLF glycans significantly reduced *Salmonella* invasion of colonic epithelial cells to levels associated with non-invasive deletion mutations [32].

The invasion of EPEC or EHEC O157:H7 into HT-29 and their derived cells definitely occurred, but the mechanism of this invasion is largely unclear, especially the greater bacterial invasion into *O*-glycosylation synthesis-inhibited HT-29 cells and core 2 *O*-glycan-deficient HT-29 cells. These data suggested that the biosynthesis of *O*-glycans residing on mucins was protective against EPEC or EHEC O157:H7 invasion into cells. In particular, in our study, we observed their invasive ability in O-glycan-abrogated HT-29 cells and core 2 mucin-type O-glycan-deficient HT-29 cells and found that EPEC or EHEC O157:H7 bacteria that invaded into intestinal epithelial cells were significantly increased. This finding might be explained by the ability of EPEC or EHEC O157:H7 to penetrate O-glycan-deficient HT-29 cells based on results from O-glycan-deficient HT-29 cells that had no attachment receptors for EPEC or EHEC O157:H7 adherence [7] and that lacked a protection barrier for intestinal epithelial cells; thus, the bacteria easily penetrated into the target cells. Although no reports in the literature have revealed that O-glycan is related to EPEC or EHEC O157:H7 invasion into intestinal epithelial cells, glycosylation changes are believed to play important roles in susceptibility to urinary tract infection and invasion of uropathogenic *E. coli* [33], and loss of core 1-, core 2- or core 3-derived *O*-glycans in animal models caused spontaneous colitis in mice [4, 34]. Further, evidence indicated that EHEC O157:H7 binds to isolated human mucus [35].

## Conclusions

Our present data confirmed that EPEC or EHEC O157:H7 invasion into HT-29 cells was related to their *O*-glycosylation status, and mucin-type core 2 *O*-glycan in HT-29 epithelial cells might be one of the pivotal structures of *O*-glycosylated chains as protection mechanisms against EPEC or EHEC O157:H7 invasion. This study provided the first evidence of carbohydrate-dependent EPEC or EHEC O157:H7 invasion into host cells and the concepts toward the design of carbohydrate-dependent inhibition of EPEC and EHEC O157:H7 invasion into human intestinal epithelial cells.

## Methods

### Materials and cell lines

EPEC E2348/69 strain (serotype: O157:H7) (EPEC) and EHEC Sakai stain (serotype: O157:H7) (EHEC O157:H7), benzyl-α-GalNAc, and HT-29, HT-29-Gal, HT-29-OBN, HT-29-Gal-OBN, C2GnT2-sh2/HT-29 and shRNA-Ctr/HT-29 cells were described previously [7].

### Lectin histochemistry

Coverslips with different cells were fixed with 4 % paraformaldehyde, rinsed with PBS several times and incubated with 0.3 % H_2_O_2_ in PBS for 30 min to block endogenous peroxidase activity. After incubation with one of the six different FITC-conjugated lectins (PNA, MAA, DBA, UEA-I, GSAII and ConA) at the concentration of 20 μg/ml or the three different biotinylated lectins (MAA, PNA, and SNA) at the concentration of 15 μg/ml for 12–14 h at room temperature (all lectins were purchased from Vector Laboratories), the coverslips were washed three times in PBS, followed by incubation with ABC (Vector Laboratories) for 60 min and washing again three times with PBS. The lectin binding site was visualized by incubating the slides with 0.05 M Tris–HCl buffer (pH 7.5) containing 0.08 % diaminobenzidine (DAB) and 0.003 % H_2_O_2_. The coverslips were lightly counterstained with hematoxylin and were examined using light microscopy. The cells stained with different FITC-conjugated lectins at the concentration of 20 μg/ml for 1 h at room temperature and counterstained with 50 µl DAPI (1:2000 in PBS) solution for 2 min at room temperature were directly observed with a Zeiss LSM 780 confocal microscope under sequential mode to avoid crosstalk, using a 40× objective, a 1.2 NA oil immersion objective and an Ar ion and HeNe lasers (488 nm excitation). The confocal image acquisition was performed so that all samples were imaged using consistent settings for laser power and detector gain.

### Bacteria invasion assay

HT-29-Gal, HT-29-Gal-OBN, HT-29, shRNA-Ctr/HT-29 and C2GnT-2-sh2/HT-29 cells were seeded and grown in different tissue culture media in 24-well tissue culture plates until confluent monolayers formed [7]. The cells were washed three times with sterilized PBS (pH 7.4) and then were cultured in an antibiotic-free cell culture medium for 2 h. After co-culturing with a range of EPEC or EHEC O157:H7 (10^5^, 10^6^, and 10^7^ CFU/0.1 mL PBS, pH 7.4)for 2 h at 37 °C, with at a multiplicity of infection (MOI) of 5:1-20:1, and washing with pre-chilled and sterilized PBS (pH 7.4) three times, the cells were cultured in 100 μg/ml gentamicin containing cell culture medium for 2 h to kill the bacteria that were adherent to ecto-intestinal epithelial cells. The cells were then washed with pre-chilled and sterilized PBS (pH 7.4) three times for 5 min per wash. The bacteria, which were released from inside the intestinal epithelial cells with 200 μl 0.25 % trypsin and 0.025 % Triton X-100 per well to rupture the cells, and they were quantified by determining the colony forming units (CFU) following the plating of serial dilutions of the bacteria on MacConkey agar with overnight incubation at 37 °C [36].

### Identification of bacterial invasion with GFP-labeled EPEC or EHEC O157:H7

The stably transformed EPEC or EHEC O157:H7 bacteria with the prokaryotic pGFP plasmid were selected with 150 μg/ml ampicillin (defined as GFP-labeled EPEC or EHEC) and were confirmed to emit green fluorescence under fluorescence microscopy, as reported previously [14]. The bacterial invasion experiments were performed, using GFP-labeled EPEC or GFP-labeled EHEC O157:H7 in HT-29-Gal; the HT-29-Gal-OBN, HT-29 or HT-29-OBN tissue culture filters housing the epithelial cell monolayers were then carefully detached from their support and mounted on coverslips, and the coverslips were then washed three times with PBS and fixed with 4 % paraformaldehyde for 30 min after co-culturing with GFP-labeled bacteria and tissue cells and further gentamicin treatment. They were then further analyzed with a Zeiss LSM 780 confocal microscope in sequential mode to avoid crosstalk between channels, with a 1.2 NA oil immersion objective and Ar ion and HeNe lasers (488 nm excitation). The confocal image acquisition was performed so that all samples were imaged using consistent settings for laser power and detector gain.

### Statistical analysis

The data are expressed as the mean ± standard deviation and were estimated by Student’s *t* test. All differences were deemed significant when p < 0.05 and very significant when p < 0.01.
